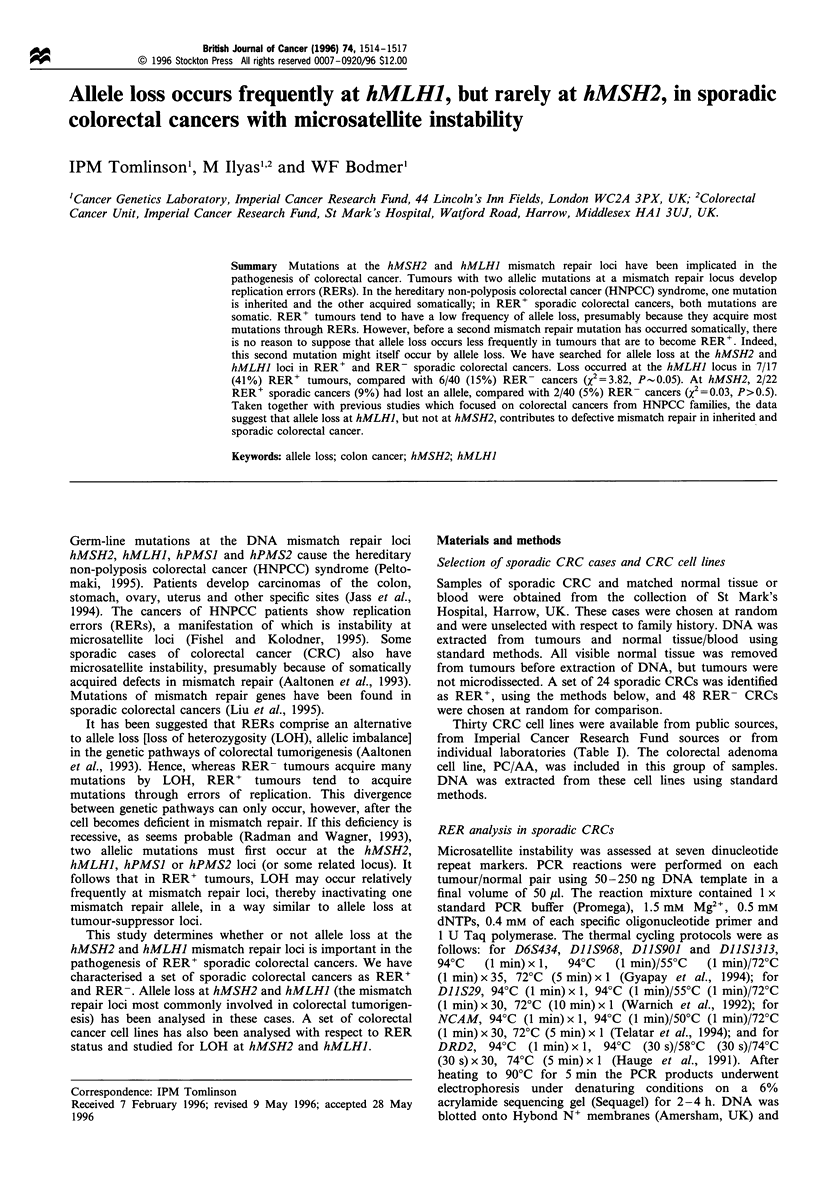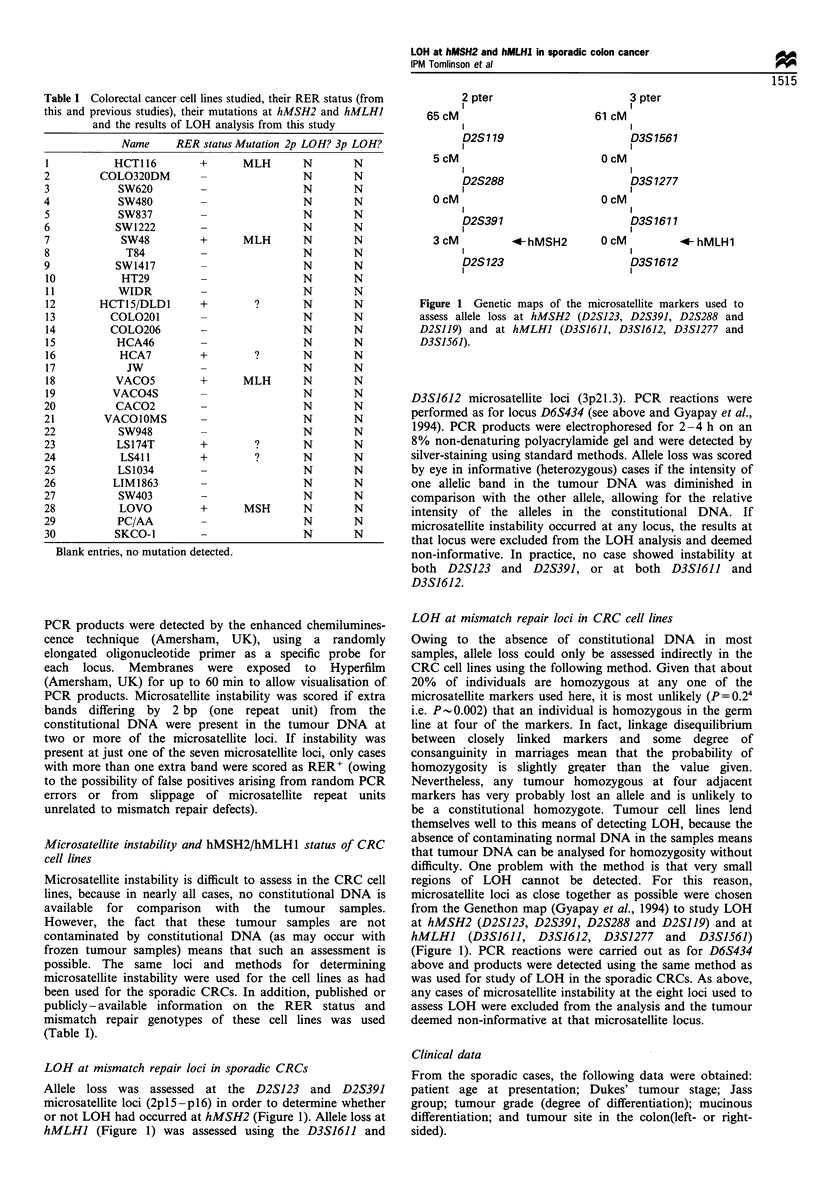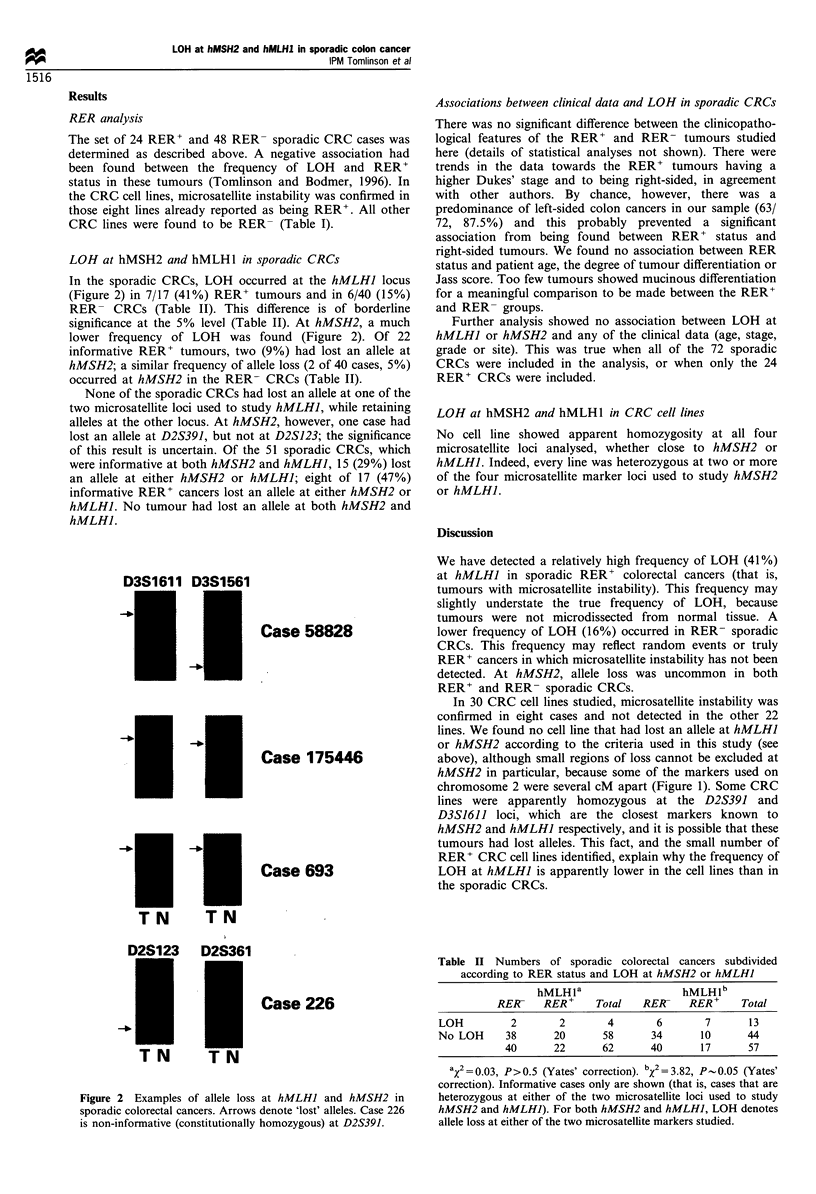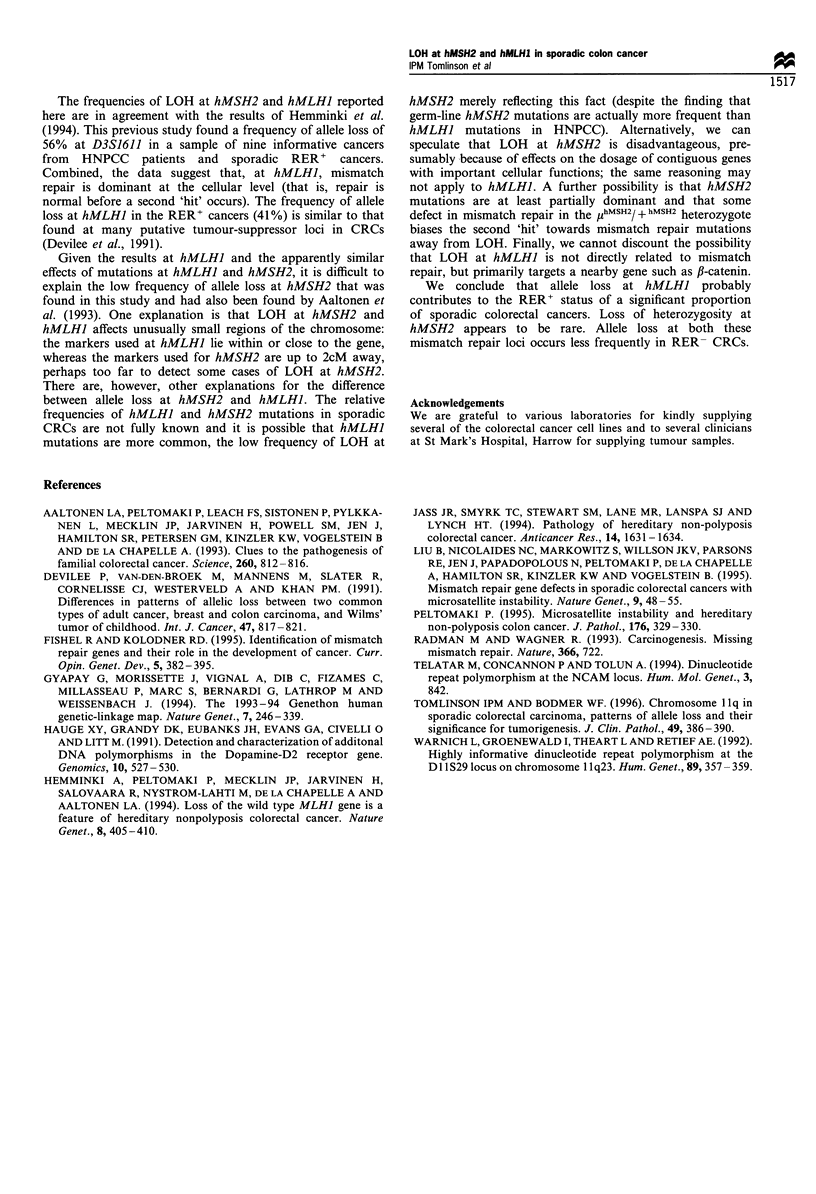# Allele loss occurs frequently at hMLH1, but rarely at hMSH2, in sporadic colorectal cancers with microsatellite instability.

**DOI:** 10.1038/bjc.1996.582

**Published:** 1996-11

**Authors:** I. P. Tomlinson, M. Ilyas, W. F. Bodmer

**Affiliations:** Cancer Genetics Laboratory, Imperial Cancer Research Fund, London, UK.

## Abstract

**Images:**


British Journal of Cancer (1996) 74, 1514–1517
© 1996 Stockton Press All rights reserved 0007–0920/96 $12.00

### Allele loss occurs frequently at *hMLH1*, but rarely at *hMSH2*, in sporadic colorectal cancers with microsatellite instability

IPM Tomlinson[1], M Ilyas[1,2] and WF Bodmer[1]

[1]*Cancer Genetics Laboratory, Imperial Cancer Research Fund, 44 Lincoln's Inn Fields, London WC2A 3PX, UK;* [2]*Colorectal Cancer Unit, Imperial Cancer Research Fund, St Mark's Hospital, Watford Road, Harrow, Middlesex HA1 3UJ, UK.*

**Summary** Mutations at the *hMSH2* and *hMLH1* mismatch repair loci have been implicated in the pathogenesis of colorectal cancer. Tumours with two allelic mutations at a mismatch repair locus develop replication errors (RERs). In the hereditary non-polyposis colorectal cancer (HNPCC) syndrome, one mutation is inherited and the other acquired somatically; in $RER^+$ sporadic colorectal cancers, both mutations are somatic. $RER^+$ tumours tend to have a low frequency of allele loss, presumably because they acquire most mutations through RERs. However, before a second mismatch repair mutation has occurred somatically, there is no reason to suppose that allele loss occurs less frequently in tumours that are to become $RER^+$. Indeed, this second mutation might itself occur by allele loss. We have searched for allele loss at the *hMSH2* and *hMLH1* loci in $RER^+$ and $RER^-$ sporadic colorectal cancers. Loss occurred at the *hMLH1* locus in 7/17 (41%) $RER^+$ tumours, compared with 6/40 (15%) $RER^-$ cancers ($\chi^2=3.82$, $P\sim0.05$). At *hMSH2*, 2/22 $RER^+$ sporadic cancers (9%) had lost an allele, compared with 2/40 (5%) $RER^-$ cancers ($\chi^2=0.03$, $P>0.5$). Taken together with previous studies which focused on colorectal cancers from HNPCC families, the data suggest that allele loss at *hMLH1*, but not at *hMSH2*, contributes to defective mismatch repair in inherited and sporadic colorectal cancer.

**Keywords:** allele loss; colon cancer; *hMSH2*; *hMLH1*

Germ-line mutations at the DNA mismatch repair loci *hMSH2*, *hMLH1*, *hPMS1* and *hPMS2* cause the hereditary non-polyposis colorectal cancer (HNPCC) syndrome (Peltomaki, 1995). Patients develop carcinomas of the colon, stomach, ovary, uterus and other specific sites (Jass *et al.*, 1994). The cancers of HNPCC patients show replication errors (RERs), a manifestation of which is instability at microsatellite loci (Fishel and Kolodner, 1995). Some sporadic cases of colorectal cancer (CRC) also have microsatellite instability, presumably because of somatically acquired defects in mismatch repair (Aaltonen *et al.*, 1993). Mutations of mismatch repair genes have been found in sporadic colorectal cancers (Liu *et al.*, 1995).

It has been suggested that RERs comprise an alternative to allele loss [loss of heterozygosity (LOH), allelic imbalance] in the genetic pathways of colorectal tumorigenesis (Aaltonen *et al.*, 1993). Hence, whereas $RER^-$ tumours acquire many mutations by LOH, $RER^+$ tumours tend to acquire mutations through errors of replication. This divergence between genetic pathways can only occur, however, after the cell becomes deficient in mismatch repair. If this deficiency is recessive, as seems probable (Radman and Wagner, 1993), two allelic mutations must first occur at the *hMSH2*, *hMLH1*, *hPMS1* or *hPMS2* loci (or some related locus). It follows that in $RER^+$ tumours, LOH may occur relatively frequently at mismatch repair loci, thereby inactivating one mismatch repair allele, in a way similar to allele loss at tumour-suppressor loci.

This study determines whether or not allele loss at the *hMSH2* and *hMLH1* mismatch repair loci is important in the pathogenesis of $RER^+$ sporadic colorectal cancers. We have characterised a set of sporadic colorectal cancers as $RER^+$ and $RER^-$. Allele loss at *hMSH2* and *hMLH1* (the mismatch repair loci most commonly involved in colorectal tumorigenesis) has been analysed in these cases. A set of colorectal cancer cell lines has also been analysed with respect to RER status and studied for LOH at *hMSH2* and *hMLH1*.

Correspondence: IPM Tomlinson
Received 7 February 1996; revised 9 May 1996; accepted 28 May 1996

### Materials and methods

### *Selection of sporadic CRC cases and CRC cell lines*

Samples of sporadic CRC and matched normal tissue or blood were obtained from the collection of St Mark's Hospital, Harrow, UK. These cases were chosen at random and were unselected with respect to family history. DNA was extracted from tumours and normal tissue/blood using standard methods. All visible normal tissue was removed from tumours before extraction of DNA, but tumours were not microdissected. A set of 24 sporadic CRCs was identified as $RER^+$, using the methods below, and 48 $RER^-$ CRCs were chosen at random for comparison.

Thirty CRC cell lines were available from public sources, from Imperial Cancer Research Fund sources or from individual laboratories (Table I). The colorectal adenoma cell line, PC/AA, was included in this group of samples. DNA was extracted from these cell lines using standard methods.

### *RER analysis in sporadic CRCs*

Microsatellite instability was assessed at seven dinucleotide repeat markers. PCR reactions were performed on each tumour/normal pair using 50–250 ng DNA template in a final volume of 50 $\mu l$. The reaction mixture contained 1 × standard PCR buffer (Promega), 1.5 mM $Mg^{2+}$, 0.5 mM dNTPs, 0.4 mM of each specific oligonucleotide primer and 1 U Taq polymerase. The thermal cycling protocols were as follows: for *D6S434*, *D11S968*, *D11S901* and *D11S1313*, 94°C (1 min) × 1, 94°C (1 min)/55°C (1 min)/72°C (1 min) × 35, 72°C (5 min) × 1 (Gyapay *et al.*, 1994); for *D11S29*, 94°C (1 min) × 1, 94°C (1 min)/55°C (1 min)/72°C (1 min) × 30, 72°C (10 min) × 1 (Warnich *et al.*, 1992); for *NCAM*, 94°C (1 min) × 1, 94°C (1 min)/50°C (1 min)/72°C (1 min) × 30, 72°C (5 min) × 1 (Telatar *et al.*, 1994); and for *DRD2*, 94°C (1 min) × 1, 94°C (30 s)/58°C (30 s)/74°C (30 s) × 30, 74°C (5 min) × 1 (Hauge *et al.*, 1991). After heating to 90°C for 5 min the PCR products underwent electrophoresis under denaturing conditions on a 6% acrylamide sequencing gel (Sequagel) for 2–4 h. DNA was blotted onto Hybond $N^+$ membranes (Amersham, UK) and

**Table I** Colorectal cancer cell lines studied, their RER status (from this and previous studies), their mutations at *hMSH2* and *hMLH1* and the results of LOH analysis from this study

| | *Name* | *RER status* | *Mutation* | *2p LOH?* | *3p LOH?* |
|---|---|---|---|---|---|
| 1 | HCT116 | + | MLH | N | N |
| 2 | COLO320DM | – | | N | N |
| 3 | SW620 | – | | N | N |
| 4 | SW480 | – | | N | N |
| 5 | SW837 | – | | N | N |
| 6 | SW1222 | – | | N | N |
| 7 | SW48 | + | MLH | N | N |
| 8 | T84 | – | | N | N |
| 9 | SW1417 | – | | N | N |
| 10 | HT29 | – | | N | N |
| 11 | WIDR | – | | N | N |
| 12 | HCT15/DLD1 | + | ? | N | N |
| 13 | COLO201 | – | | N | N |
| 14 | COLO206 | – | | N | N |
| 15 | HCA46 | – | | N | N |
| 16 | HCA7 | + | ? | N | N |
| 17 | JW | – | | N | N |
| 18 | VACO5 | + | MLH | N | N |
| 19 | VACO4S | – | | N | N |
| 20 | CACO2 | – | | N | N |
| 21 | VACO10MS | – | | N | N |
| 22 | SW948 | – | | N | N |
| 23 | LS174T | + | ? | N | N |
| 24 | LS411 | + | ? | N | N |
| 25 | LS1034 | – | | N | N |
| 26 | LIM1863 | – | | N | N |
| 27 | SW403 | – | | N | N |
| 28 | LOVO | + | MSH | N | N |
| 29 | PC/AA | – | | N | N |
| 30 | SKCO-1 | – | | N | N |

Blank entries, no mutation detected.

PCR products were detected by the enhanced chemiluminescence technique (Amersham, UK), using a randomly elongated oligonucleotide primer as a specific probe for each locus. Membranes were exposed to Hyperfilm (Amersham, UK) for up to 60 min to allow visualisation of PCR products. Microsatellite instability was scored if extra bands differing by 2 bp (one repeat unit) from the constitutional DNA were present in the tumour DNA at two or more of the microsatellite loci. If instability was present at just one of the seven microsatellite loci, only cases with more than one extra band were scored as RER$^+$ (owing to the possibility of false positives arising from random PCR errors or from slippage of microsatellite repeat units unrelated to mismatch repair defects).

### *Microsatellite instability and* hMSH2/hMLH1 *status of CRC cell lines*

Microsatellite instability is difficult to assess in the CRC cell lines, because in nearly all cases, no constitutional DNA is available for comparison with the tumour samples. However, the fact that these tumour samples are not contaminated by constitutional DNA (as may occur with frozen tumour samples) means that such an assessment is possible. The same loci and methods for determining microsatellite instability were used for the cell lines as had been used for the sporadic CRCs. In addition, published or publicly-available information on the RER status and mismatch repair genotypes of these cell lines was used (Table I).

### *LOH at mismatch repair loci in sporadic CRCs*

Allele loss was assessed at the *D2S123* and *D2S391* microsatellite loci (2p15–p16) in order to determine whether or not LOH had occurred at *hMSH2* (Figure 1). Allele loss at *hMLH1* (Figure 1) was assessed using the *D3S1611* and *D3S1612* microsatellite loci (3p21.3). PCR reactions were performed as for locus *D6S434* (see above and Gyapay *et al.*, 1994). PCR products were electrophoresed for 2–4 h on an 8% non-denaturing polyacrylamide gel and were detected by silver-staining using standard methods. Allele loss was scored by eye in informative (heterozygous) cases if the intensity of one allelic band in the tumour DNA was diminished in comparison with the other allele, allowing for the relative intensity of the alleles in the constitutional DNA. If microsatellite instability occurred at any locus, the results at that locus were excluded from the LOH analysis and deemed non-informative. In practice, no case showed instability at both *D2S123* and *D2S391*, or at both *D3S1611* and *D3S1612*.

**Figure 1** Genetic maps of the microsatellite markers used to assess allele loss at *hMSH2* (*D2S123*, *D2S391*, *D2S288* and *D2S119*) and at *hMLH1* (*D3S1611*, *D3S1612*, *D3S1277* and *D3S1561*).

### *LOH at mismatch repair loci in CRC cell lines*

Owing to the absence of constitutional DNA in most samples, allele loss could only be assessed indirectly in the CRC cell lines using the following method. Given that about 20% of individuals are homozygous at any one of the microsatellite markers used here, it is most unlikely ($P = 0.2^4$ i.e. $P \sim 0.002$) that an individual is homozygous in the germ line at four of the markers. In fact, linkage disequilibrium between closely linked markers and some degree of consanguinity in marriages mean that the probability of homozygosity is slightly greater than the value given. Nevertheless, any tumour homozygous at four adjacent markers has very probably lost an allele and is unlikely to be a constitutional homozygote. Tumour cell lines lend themselves well to this means of detecting LOH, because the absence of contaminating normal DNA in the samples means that tumour DNA can be analysed for homozygosity without difficulty. One problem with the method is that very small regions of LOH cannot be detected. For this reason, microsatellite loci as close together as possible were chosen from the Genethon map (Gyapay *et al.*, 1994) to study LOH at *hMSH2* (*D2S123*, *D2S391*, *D2S288* and *D2S119*) and at *hMLH1* (*D3S1611*, *D3S1612*, *D3S1277* and *D3S1561*) (Figure 1). PCR reactions were carried out as for *D6S434* above and products were detected using the same method as was used for study of LOH in the sporadic CRCs. As above, any cases of microsatellite instability at the eight loci used to assess LOH were excluded from the analysis and the tumour deemed non-informative at that microsatellite locus.

### *Clinical data*

From the sporadic cases, the following data were obtained: patient age at presentation; Dukes' tumour stage; Jass group; tumour grade (degree of differentiation); mucinous differentiation; and tumour site in the colon(left- or right-sided).

### Results

### *RER analysis*

The set of 24 $RER^+$ and 48 $RER^-$ sporadic CRC cases was determined as described above. A negative association had been found between the frequency of LOH and $RER^+$ status in these tumours (Tomlinson and Bodmer, 1996). In the CRC cell lines, microsatellite instability was confirmed in those eight lines already reported as being $RER^+$. All other CRC lines were found to be $RER^-$ (Table I).

### *LOH at* hMSH2 *and* hMLH1 *in sporadic CRCs*

In the sporadic CRCs, LOH occurred at the *hMLH1* locus (Figure 2) in 7/17 (41%) $RER^+$ tumours and in 6/40 (15%) $RER^-$ CRCs (Table II). This difference is of borderline significance at the 5% level (Table II). At *hMSH2*, a much lower frequency of LOH was found (Figure 2). Of 22 informative $RER^+$ tumours, two (9%) had lost an allele at *hMSH2*; a similar frequency of allele loss (2 of 40 cases, 5%) occurred at *hMSH2* in the $RER^-$ CRCs (Table II).

None of the sporadic CRCs had lost an allele at one of the two microsatellite loci used to study *hMLH1*, while retaining alleles at the other locus. At *hMSH2*, however, one case had lost an allele at *D2S391*, but not at *D2S123*; the significance of this result is uncertain. Of the 51 sporadic CRCs, which were informative at both *hMSH2* and *hMLH1*, 15 (29%) lost an allele at either *hMSH2* or *hMLH1*; eight of 17 (47%) informative $RER^+$ cancers lost an allele at either *hMSH2* or *hMLH1*. No tumour had lost an allele at both *hMSH2* and *hMLH1*.

**Figure 2** Examples of allele loss at *hMLH1* and *hMSH2* in sporadic colorectal cancers. Arrows denote 'lost' alleles. Case 226 is non-informative (constitutionally homozygous) at *D2S391*.

### *Associations between clinical data and LOH in sporadic CRCs*

There was no significant difference between the clinicopathological features of the $RER^+$ and $RER^-$ tumours studied here (details of statistical analyses not shown). There were trends in the data towards the $RER^+$ tumours having a higher Dukes' stage and to being right-sided, in agreement with other authors. By chance, however, there was a predominance of left-sided colon cancers in our sample (63/72, 87.5%) and this probably prevented a significant association from being found between $RER^+$ status and right-sided tumours. We found no association between RER status and patient age, the degree of tumour differentiation or Jass score. Too few tumours showed mucinous differentiation for a meaningful comparison to be made between the $RER^+$ and $RER^-$ groups.

Further analysis showed no association between LOH at *hMLH1* or *hMSH2* and any of the clinical data (age, stage, grade or site). This was true when all of the 72 sporadic CRCs were included in the analysis, or when only the 24 $RER^+$ CRCs were included.

### *LOH at* hMSH2 *and* hMLH1 *in CRC cell lines*

No cell line showed apparent homozygosity at all four microsatellite loci analysed, whether close to *hMSH2* or *hMLH1*. Indeed, every line was heterozygous at two or more of the four microsatellite marker loci used to study *hMSH2* or *hMLH1*.

### Discussion

We have detected a relatively high frequency of LOH (41%) at *hMLH1* in sporadic $RER^+$ colorectal cancers (that is, tumours with microsatellite instability). This frequency may slightly understate the true frequency of LOH, because tumours were not microdissected from normal tissue. A lower frequency of LOH (16%) occurred in $RER^-$ sporadic CRCs. This frequency may reflect random events or truly $RER^+$ cancers in which microsatellite instability has not been detected. At *hMSH2*, allele loss was uncommon in both $RER^+$ and $RER^-$ sporadic CRCs.

In 30 CRC cell lines studied, microsatellite instability was confirmed in eight cases and not detected in the other 22 lines. We found no cell line that had lost an allele at *hMLH1* or *hMSH2* according to the criteria used in this study (see above), although small regions of loss cannot be excluded at *hMSH2* in particular, because some of the markers used on chromosome 2 were several cM apart (Figure 1). Some CRC lines were apparently homozygous at the *D2S391* and *D3S1611* loci, which are the closest markers known to *hMSH2* and *hMLH1* respectively, and it is possible that these tumours had lost alleles. This fact, and the small number of $RER^+$ CRC cell lines identified, explain why the frequency of LOH at *hMLH1* is apparently lower in the cell lines than in the sporadic CRCs.

**Table II** Numbers of sporadic colorectal cancers subdivided according to RER status and LOH at *hMSH2* or *hMLH1*

| | hMLH1[a] | | | hMLH1[b] | | |
|---|---|---|---|---|---|---|
| | *$RER^-$* | *$RER^+$* | *Total* | *$RER^-$* | *$RER^+$* | *Total* |
| LOH | 2 | 2 | 4 | 6 | 7 | 13 |
| No LOH | 38 | 20 | 58 | 34 | 10 | 44 |
| | 40 | 22 | 62 | 40 | 17 | 57 |

[a]$\chi^2 = 0.03$, $P > 0.5$ (Yates' correction). [b]$\chi^2 = 3.82$, $P \sim 0.05$ (Yates' correction). Informative cases only are shown (that is, cases that are heterozygous at either of the two microsatellite loci used to study *hMSH2* and *hMLH1*). For both *hMSH2* and *hMLH1*, LOH denotes allele loss at either of the two microsatellite markers studied.

The frequencies of LOH at *hMSH2* and *hMLH1* reported here are in agreement with the results of Hemminki *et al.* (1994). This previous study found a frequency of allele loss of 56% at *D3S1611* in a sample of nine informative cancers from HNPCC patients and sporadic $RER^+$ cancers. Combined, the data suggest that, at *hMLH1*, mismatch repair is dominant at the cellular level (that is, repair is normal before a second 'hit' occurs). The frequency of allele loss at *hMLH1* in the $RER^+$ cancers (41%) is similar to that found at many putative tumour-suppressor loci in CRCs (Devilee *et al.*, 1991).

Given the results at *hMLH1* and the apparently similar effects of mutations at *hMLH1* and *hMSH2*, it is difficult to explain the low frequency of allele loss at *hMSH2* that was found in this study and had also been found by Aaltonen *et al.* (1993). One explanation is that LOH at *hMSH2* and *hMLH1* affects unusually small regions of the chromosome: the markers used at *hMLH1* lie within or close to the gene, whereas the markers used for *hMSH2* are up to 2cM away, perhaps too far to detect some cases of LOH at *hMSH2*. There are, however, other explanations for the difference between allele loss at *hMSH2* and *hMLH1*. The relative frequencies of *hMLH1* and *hMSH2* mutations in sporadic CRCs are not fully known and it is possible that *hMLH1* mutations are more common, the low frequency of LOH at *hMSH2* merely reflecting this fact (despite the finding that germ-line *hMSH2* mutations are actually more frequent than *hMLH1* mutations in HNPCC). Alternatively, we can speculate that LOH at *hMSH2* is disadvantageous, presumably because of effects on the dosage of contiguous genes with important cellular functions; the same reasoning may not apply to *hMLH1*. A further possibility is that *hMSH2* mutations are at least partially dominant and that some defect in mismatch repair in the $\mu^{hMSH2}/+^{hMSH2}$ heterozygote biases the second 'hit' towards mismatch repair mutations away from LOH. Finally, we cannot discount the possibility that LOH at *hMLH1* is not directly related to mismatch repair, but primarily targets a nearby gene such as $\beta$-catenin.

We conclude that allele loss at *hMLH1* probably contributes to the $RER^+$ status of a significant proportion of sporadic colorectal cancers. Loss of heterozygosity at *hMSH2* appears to be rare. Allele loss at both these mismatch repair loci occurs less frequently in $RER^-$ CRCs.

**Acknowledgements**

We are grateful to various laboratories for kindly supplying several of the colorectal cancer cell lines and to several clinicians at St Mark's Hospital, Harrow for supplying tumour samples.

### References

AALTONEN LA, PELTOMAKI P, LEACH FS, SISTONEN P, PYLKKANEN L, MECKLIN JP, JARVINEN H, POWELL SM, JEN J, HAMILTON SR, PETERSEN GM, KINZLER KW, VOGELSTEIN B AND DE LA CHAPELLE A. (1993). Clues to the pathogenesis of familial colorectal cancer. *Science*, **260**, 812–816.

DEVILEE P, VAN-DEN-BROEK M, MANNENS M, SLATER R, CORNELISSE CJ, WESTERVELD A AND KHAN PM. (1991). Differences in patterns of allelic loss between two common types of adult cancer, breast and colon carcinoma, and Wilms' tumor of childhood. *Int. J. Cancer*, **47**, 817–821.

FISHEL R AND KOLODNER RD. (1995). Identification of mismatch repair genes and their role in the development of cancer. *Curr. Opin. Genet. Dev.*, **5**, 382–395.

GYAPAY G, MORISSETTE J, VIGNAL A, DIB C, FIZAMES C, MILLASSEAU P, MARC S, BERNARDI G, LATHROP M AND WEISSENBACH J. (1994). The 1993–94 Genethon human genetic-linkage map. *Nature Genet.*, **7**, 246–339.

HAUGE XY, GRANDY DK, EUBANKS JH, EVANS GA, CIVELLI O AND LITT M. (1991). Detection and characterization of additonal DNA polymorphisms in the Dopamine-D2 receptor gene. *Genomics*, **10**, 527–530.

HEMMINKI A, PELTOMAKI P, MECKLIN JP, JARVINEN H, SALOVAARA R, NYSTROM-LAHTI M, DE LA CHAPELLE A AND AALTONEN LA. (1994). Loss of the wild type *MLH1* gene is a feature of hereditary nonpolyposis colorectal cancer. *Nature Genet.*, **8**, 405–410.

JASS JR, SMYRK TC, STEWART SM, LANE MR, LANSPA SJ AND LYNCH HT. (1994). Pathology of hereditary non-polyposis colorectal cancer. *Anticancer Res.*, **14**, 1631–1634.

LIU B, NICOLAIDES NC, MARKOWITZ S, WILLSON JKV, PARSONS RE, JEN J, PAPADOPOLOUS N, PELTOMAKI P, DE LA CHAPELLE A, HAMILTON SR, KINZLER KW AND VOGELSTEIN B. (1995). Mismatch repair gene defects in sporadic colorectal cancers with microsatellite instability. *Nature Genet.*, **9**, 48–55.

PELTOMAKI P. (1995). Microsatellite instability and hereditary non-polyposis colon cancer. *J. Pathol.*, **176**, 329–330.

RADMAN M AND WAGNER R. (1993). Carcinogenesis. Missing mismatch repair. *Nature*, **366**, 722.

TELATAR M, CONCANNON P AND TOLUN A. (1994). Dinucleotide repeat polymorphism at the NCAM locus. *Hum. Mol. Genet.*, **3**, 842.

TOMLINSON IPM AND BODMER WF. (1996). Chromosome 11q in sporadic colorectal carcinoma, patterns of allele loss and their significance for tumorigenesis. *J. Clin. Pathol.*, **49**, 386–390.

WARNICH L, GROENEWALD I, THEART L AND RETIEF AE. (1992). Highly informative dinucleotide repeat polymorphism at the D11S29 locus on chromosome 11q23. *Hum. Genet.*, **89**, 357–359.